# Ultrasound‐Responsive Oxygen‐Carrying Pollen for Enhancing Chemo‐Sonodynamic Therapy of Breast Cancer

**DOI:** 10.1002/advs.202300456

**Published:** 2023-05-16

**Authors:** Baojie Wen, Danqing Huang, Chuanhui Song, Jingyang Shan, Yuanjin Zhao

**Affiliations:** ^1^ Department of Ultrasound Institute of Translational Medicine Nanjing Drum Tower Hospital Affiliated Hospital of Medicine School Nanjing University Nanjing 210008 China; ^2^ State Key Laboratory of Bioelectronics School of Biological Science and Medical Engineering Southeast University Nanjing 210096 China

**Keywords:** chemo‐sonodynamic therapy, drug delivery, microcarrier, oxygen carrying, pollen

## Abstract

The tumor‐suppressing efficacy of either chemotherapeutics or gaseous drugs has been confirmed in treating the triple negative breast cancer (TNBC), while the efficacy of single treatment is usually dissatisfactory. Herein, a novel ultrasound responsive natural pollen delivery system is presented to simultaneously load chemotherapeutics and gaseous drugs for synergistic treatment of TNBC. The hollow structure of pollen grains carries oxygen‐enriched perfluorocarbon (PFC), and the porous spinous process structure adsorbs the chemotherapeutic drug doxorubicin (DOX) (PO/D‐PGs). Ultrasound can trigger the oxygen release from PFC and excite DOX, which is not only a chemotherapeutic but also a sonosensitizer, to realize chemo‐sonodynamic therapy. The PO/D‐PGs are demonstrated to effectively enhance oxygen concentration and increase the production of reactive oxygen species in the presence of low‐intensity ultrasound, synergistically enhancing the tumor killing ability. Thus, the synergistic therapy based on ultrasound‐facilitated PO/D‐PGs significantly enhances the antitumor effect in the mouse TNBC model. It is believed that the proposed natural pollen cross‐state microcarrier can be used as an effective strategy to enhance chemo‐sonodynamic therapy for TNBC.

## Graphical abstract

The hollow structure of pollen grains carries oxygen‐enriched perfluorocarbon (PFC), and the porous spinous process structure adsorbs the chemotherapeutic drug doxorubicin (DOX) (PO/D‐PGs). Ultrasound can trigger the oxygen release from PFC and excite DOX to realize chemo‐sonodynamic therapy. The synergistic therapy based on ultrasound‐facilitated PO/D‐PGs can significantly enhance the antitumor effect in breast cancer.

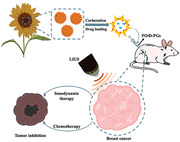

## 1 Introduction

Breast cancer is currently the leading cause of female morbidity and mortality worldwide with increasing incidence.^[^ ^1^ ^]^ Depending on the type of breast cancer, clinicians choose different treatment strategies. The TNBC is the most difficult to treat due to its lack of human epidermal growth factor receptor, estrogen receptor, and progesterone receptor expression, which usually serve as therapeutic targets for breast cancer.^[^ ^2^, ^3^ ^]^ By far the most effective treatment strategy for TNBC is systematic chemotherapy, such as intravenously administrating doxorubicin (DOX). Besides, some gaseous therapies have been explored and elucidated to have effect on inhibiting tumors.^[^ ^4^, ^5^ ^]^ For example, since the hypoxic microenvironment in TNBC increases drug resistance and promotes malignancy, the employment of oxygen can significantly contribute to the treatment of TNBC.^[^ ^6^, ^7^ ^]^ However, the effectiveness of single drug employment is relatively limited, and systematic administration of chemotherapeutics usually causes numerous side effects.^[^ ^8^, ^9^ ^]^ Although some delivery systems have been developed to realize multiple and local drug delivery, the codelivery of chemotherapeutics and gases are few reported.^[^ ^3^, ^10^ ^]^ In addition, the local release of drugs from existing delivery systems is often uncontrollable, resulting in nonoptimal therapeutic efficacy.

**Figure 1:**
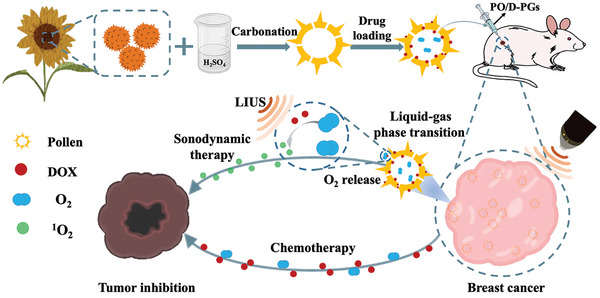
Schematic diagram of PO/D‐PGs in a mouse model of breast cancer. Ultrasound‐responsive pollen delivery system capable of delivering oxygen and chemotherapeutics simultaneously for enhancing chemo‐sonodynamic therapy of TNBC through local injection.

In this paper, we present an ultrasound‐responsive pollen delivery system capable of delivering oxygen and chemotherapeutics simultaneously for treating TNBC through local injection, as schemed in **Figure** **1**. Natural pollens have been employed in many biomedical applications.^[^ ^11^, ^12^ ^]^ According to the various morphologies of pollens from different species, their potential application value has been constantly explored. Take the pollens of sunflowers for instance, the complex three‐dimensional (3D) structure with spikes and nanopores on their surface makes them potential carriers for a variety of molecular drugs.^[^ ^13^, ^14^ ^]^ Interestingly, after being carbonized, the sunflower pollens show a unique hollow structure, making them natural drug encapsulation platforms.^[^ ^15^, ^16^ ^]^ Therefore, sunflower pollens exhibit great potential in multidrug delivery. In contrast, as a biocompatible mechanical wave, ultrasound has shown excellent performance in triggering drug release even in deep tissue.^[^ ^17^, ^18^ ^]^ Despite the tremendous achievements of ultrasound‐responsive drug release systems, the employment of pollens to carry both chemotherapeutics and therapeutic gas for ultrasound triggered therapy has not been reported.

Herein, we used hollow spiked pollens to codelivery DOX and oxygen for ultrasound‐triggered oxygen release and sonodynamic therapy (SDT) in treating TNBC. The sunflower pollens received deesterification, chemical hydrolysis, and acid treatment To achieve porous and hollow structures.^[^ ^19^, ^20^ ^]^ The distinctive micro‐structures imparted the pollens with adsorption capacity for drug delivery.^[^ ^15^, ^21^ ^]^ By infiltrating oxygen‐enriched perfluorocarbon (PFC) liquid into the core of pollen and loading DOX onto the surface, a pollen delivery system coloading oxygen and DOX (PO/D‐PGs) could be prepared. DOX is not only a chemotherapeutic but also a sonosensitizer for SDT.^[^ ^22^ ^]^ Meanwhile, PFC is a kind of biofavorable substance that can undergo liquid‐to‐gas phase transition under the trigger of ultrasound.^[^ ^23^ ^]^ We demonstrated that ultrasound could effectively trigger the PO/D‐PGs to release oxygen and produce reactive oxygen species (ROS) through SDT. In vitro experiments verified that the released oxygen contributed to the reversion of tumor hypoxia, increased the ROS production and improved the antitumor efficacy of DOX. Moreover, the ultrasound‐facilitated synergistic treatment showed outstanding antitumor efficacy in mice with TNBC. These results indicated the multikind drug codelivery capacity and ultrasound‐responsiveness of pollen‐based delivery system, showing great potential in combinational tumor treatment.

## 2 Results and Discussions

**Figure 2:**
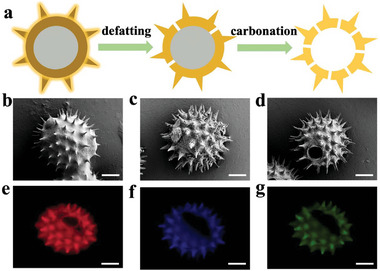
Method of preparing and characterizing of pollen grains (PGs). a) Describe the procedure for preparing PGs. b) SEM image of the water‐treated pollen. c) SEM image of the defatted pollen. d) SEM image of the defatted sunflower pollen treated with concentrated sulfuric acid. e‐g) Fluorescence images of acid‐lysed pollen obtained by fluorescence microscopy. b–g) Scale bars are 10 µm in (b)–(g).

In the experiments of this study, natural microcarriers pollen grains (PGs) were prepared from the pollen of sunflower plants, and the whole fabrication process is schemed in **Figure** **2**a. First, sunflower pollens were washed with water for several times, and the scanning electron microscopy (SEM) image indicated the surface morphology of sunflower PGs after water washing (Figure 2b). Then, acetone and ether were applied to de‐esterify the sunflower PGs, and the surface morphology of the de‐esterified sunflower PGs is shown in Figure 2c. Finally, in order to obtain hollow spiny microcyst structure, the de‐esterified pollens were dehydrated and carbonized, and the surface morphology of the de‐esterified PGs after sulfuric acid treatment is shown in Figure 2d. Because fluorophores like phenolic compounds and carotenoids are present, the defatted PGs have strong self‐fluorescence.^[^ ^13^, ^24^ ^]^ Therefore, we examined sunflower PGs under a fluorescence microscope. We found that PGs treated with deesterification and carbonization still retained strong fluorescence. The carbonized PGs still emitted red (Figure 2e), blue (Figure 2f), and green fluorescence (Figure 2g). The high temperature calcination (300 °C, 6 h) treatment effectively removed the intracytoplasmic contents, causing significant fluorescence bleaching in the periphery and lumen (Figure S1a,b, Supporting Information). After carbonization and calcination, PGs have multiple pores opening inside and on their surfaces, while maintaining their structural integrity and allowing for drug loading (Figure S1c, Supporting Information). The chemotherapeutic drug DOX can be adsorbed onto the surface of the complex 3D structure of pollen grains and on the spines, and then the oxygen‐enriched 1,1,1,2,3,4,4,5,5,5‐decafluoropentane, which is a kind of PFC liquid, is wrapped in the cavity of the pollen grains with an aqueous solution. Notably, DOX is also a small molecule sonosensitizer, allowing to construction a novel multifunctional natural microcarrier system for synergy‐enhanced chemotherapy‐SDT cancer therapy.^[^ ^22^, ^25^ ^]^

**Figure 3:**
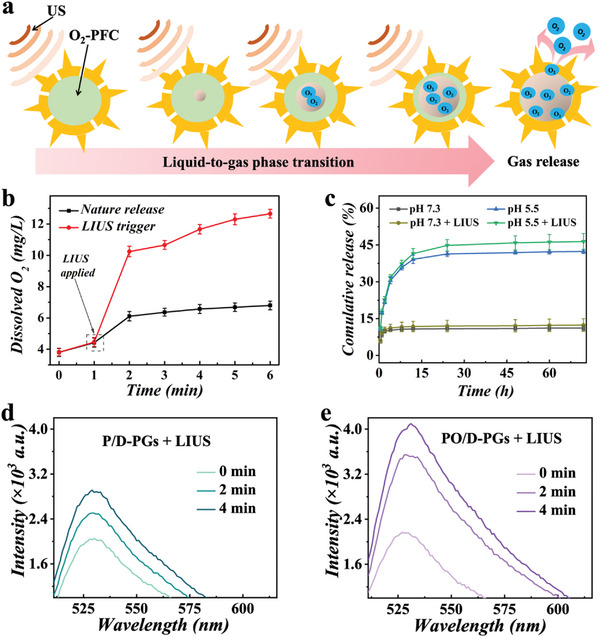
The ability of pollen grains to delivery oxygen and the phase transition of PFC. a) Principal scheme of LIUS‐triggered liquid‐gas phase transition and oxygen release from PFC encapsulated with pollen grains. b) Dissolved oxygen concentration variations for passive waiver of oxygen‐enriched PFC and LIUS‐triggered release (*n* = 3). c) The effect of LIUS and pH on the controlled release of drugs (*n* = 3). d) Fluorescence intensity curves of SOSG and pollen delivery system coloading PFC and DOX (P/D‐PGs) mixed solutions after different intervals of LIUS irradiation. e) Fluorescence intensity curves of SOSG and PO/D‐PGs mixed solutions after different intervals of LIUS irradiation.

PFC is an inert compound that exists in liquid form at normal barometric pressure and room temperature. PFC has a remarkable oxygen solubility, that is twenty times that of water.^[^ ^23^ ^]^ Due to its biocompatibility, PFC has been utilized in clinical settings for decades. Because of the hydrophobicity of PFC, the PFC droplets were encapsulated in the pollen cavity, providing Laplacian pressure between the cavity and the external environment, so that PFC can be kept as a stable liquid.^[^ ^26^, ^27^ ^]^ Additionally, it is stimulated by the low boiling point from PFC, allowing to trigger the release of the payload. Therefore, when the PFC is encapsulated, with a stimulating outward interferes at the same time, ultrasonic irradiation induces vaporization of the droplets and releases them into the external environment.^[^ ^28^ ^]^ After about 20 s of low intensity ultrasound (LIUS) application, the partially encapsulated PFC began to switch from liquid to gas, with bubbles forming as the PFC kept evaporating due to the constant LIUS stimulation (**Figure** **3**a). Pollens containing oxygen PFC nuclei were stored in 37 °C phosphate buffered saline (PBS) to better simulate the in vivo environment. The 1.0 MHz and 1.5 W cm^−2^ of LIUS was utilized to clarify the phase transformation of ultrasound‐triggered PFC with a load of oxygen and release capacity from pollen microcarriers. Pollens containing oxygen‐enriched PFC were added to two beakers of 37 °C (PBS), one of which was treated with LIUS and the other not. The surface of PBS was added by the liquid paraffin, leading it to separate from the normoxic environment. At various time intervals, the level of dissolved oxygen in PBS was measured. From Figure 3b presented, the oxygen content increased slowly and reached equilibrium in the untreated group, while it increased rapidly in the LIUS group after the application of LIUS. After stimulating LIUS by 1 min, the level of oxygen dissolution arrived at 10.2 mg L^−1^ from the LIUS‐treated group. Oxygen release from the PFC was also achieved by passive diffusion driven by oxygen concentration gradients, but its release efficiency was lower. Based on the LIUS existed, the PFC generates a liquid to gas transformation, as a result oxygen releasing. More efficiently than passive diffusion driven by gradients in oxygen concentration, the fluid‐to‐gas phase transition can aid in releasing dissolving oxygen.^[^ ^5^, ^29^ ^]^ Therefore, we evaluated the oxygen loading and release performance of the PFC and verified the efficiency of applying LIUS to facilitate the release of oxygen from the PFC, which is necessary for downstream experiments.

The hollow and unique spiky porous structure of the carbonized sunflower pollen grains makes it a potential natural drug carrier material.^[^ ^30^, ^31^ ^]^ This study, using DOX as an anticancer medicine to assess its loading efficiency. The calculation is based on the DOX at 480 nm position of characteristic absorbance. The loading efficiency in DOX in DOX‐PGS was 19.08%. The ideal loading efficiency and stability of sunflower pollen laid the foundation for the development of downstream experiments. We then studied drug release behavior in PBS buffers with varying values of pH (7.3 and 5.5). As shown in Figure 3c, with the same effect of LIUS, a lower pH accelerated the drug release, i.e., the acidic microenvironment of the tumor could promote the drug release. At the same pH, the administration of LIUS did not significantly promote DOX release. The smaller pH may have accelerated the release of DOX because it may have slowed down the electrostatic adsorption among pollen grains and DOX molecules.^[^ ^32^, ^33^ ^]^

Analyzing the PO/D‐PGs forced on their ability to carry and release oxygen, proved that it is important to provide a better oxygen supply environment to future verify the feasibility of ROS generation. Although the mechanism function of SDT requires future studying, the oxidation caused by oxygen radicals throughout acoustic dynamics has been demonstrated.^[^ ^34^ ^]^ An acoustic‐thermal effect resulting from the cavitation effect from ultrasound excitation, particularly the acoustic sensitizer would achieve the peak energy level by stimulating acoustic energy.^[^ ^35^ ^]^ The acoustic sensitizer back to the base state, the energy is transferred to neighboring oxygen molecules and subsequently generates ROS, specifically ^1^O_2_, which destroys cells through peroxidation. To evaluate in vitro ^1^O_2_ production, we employed single linear state oxygen sensor green (SOSG), which is highly fluorescent once conversing of ^1^O_2_ . The fluorescence signal intensity of SOSG showed an increasing trend with increasing ultrasound irradiation time (Figure 3d), indicating that the PO/D‐PGs system could generate ^1^O_2_ under the irradiation of LIUS. In addition, we noticed a dramatic increase within the yield of ^1^O_2_ around the existence of PO (Figure 3e), which was due to the promotion of oxygen release from PO/D‐PG under the LIUS environment, thus providing sufficient oxygen for ^1^O_2_ production. We set up a control experiment under the same conditions, and the control group was a mixture of pure water and SOSG (Figure S2, Supporting Information).

**Figure 4:**
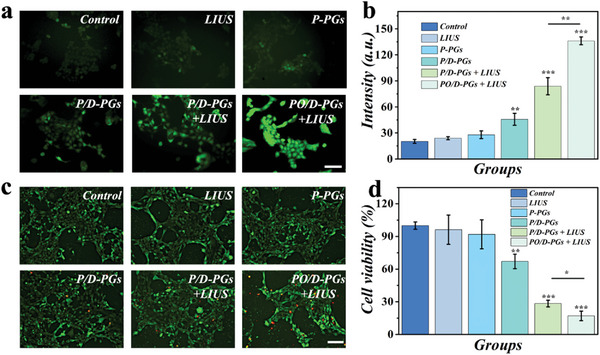
Cytotoxicity of pollen microcarriers on 4T1 cells. a) Images of ROS fluorescence intensity of each group of 4T1 cells. b) The fluorescence intensity quantification of DCFH‐DA from confocal microscopy images (*n* = 3). c) Live/dead staining images of 4T1 cells in different cell groups. d) Cell viability of 4T1 cells in different cell groups (*n* = 6). Data in (b) and (d) are showed as mean ± SD. *p < 0.05, **p < 0.01, ***p < 0.001, using the two‐sided student's *t* test. Scale bars are 100 µm in (a) and (c).

Prior to the cell experiments, the biological compatibility of the materials was evaluated. 3T3 cells were coincubated with pollen‐containing PFC encapsulation (P‐PGs) (Figure S3, Supporting Information). Images of live/dead staining and results from cell counting kit‐8 (CCK‐8) showed that PGs and PFC were nontoxic. Breast cancer cell line 4T1 cells were used for downstream cell experiments. Initially, the production of intracellular ROS was analyzed using the ROS probe DCFH‐DA. In presence of ROS, the probe can be transferred to 2,7‐dichlorofluorescein (DCF), which emits green fluorescence. There were six groups: the control group, LIUS group, P‐PGs group, PFC/DOX‐PGs (P/D‐PGs) group, P/D‐PGs + LIUS group, and PO/D‐PGs + LIUS group. Before treatment, the probe DCFH‐DA was included in the medium. Using confocal fluorescence microscopy, 4T1 cells treated with LIUS were observed afterwards (**Figure** **4**a). The average intensity of fluorescence can be indicated that PO/D‐PGs can resultful stimulate ROS production in the intracellular. As shown in Figure 4b, the PO/D‐PGs group showed stronger green fluorescence than the P/D‐PGs group during ultrasound irradiation, demonstrating that oxygen synergistically enhanced the production of ROS. To verify the hydroxyl radical (•OH) in solution irradiated by LIUS, electron spin resonance (ESR) spectroscopy and the •OH sensitive trapping agents 5,5‐dimethyl‐1‐pyrroline *N*‐oxide (DMPO) were used. The curves in Figure S4 (Supporting Information) showed the characteristics of the DMPO−•OH spin adduct from Control, P/D‐PGs, and PO/D‐PGs solution under LIUS irradiation. The characteristic peak of PO/D‐PGs was significantly stronger than that of P/D‐PGs.

Then, additional study groups were established to confirm the cytotoxicity and ability of ameliorate hypoxia of the PO/D‐PGs + LIUS system. 4T1 cells were cultured in a standard medium as part of the control group. Cells in the LIUS group were dealt with LIUS for 2 min in the standard media. Cells in the P‐PGs and P/D‐PGs groups were coincubated with P‐PGs and P/D‐PGs, respectively. The 4T1 cells in the P/D‐PGs + LIUS and PO/D‐PGs + LIUS groups were coincubated with their respective pollen grains for 40 min, and then LIUS was implemented for 2 min. As shown in Figure 4c, the morphological identification of 4T1 cells in control, LIUS, and P‐PGs groups were normal, indicating that LIUS and pollen grains were biosafe. All three latter groups exhibited higher cytotoxicity due to the release of DOX. Notably, the PO/D‐PGs + LIUS group showed more apoptosis compared to the P/D‐PGs + LIUS group. Fluorescence images of both living and dead things support CCK‐8 studies proving that cell viability was significantly lower in the PO/D‐PGs + LIUS group (17.09 ± 4.39%) than that in the P/D‐PGs + LIUS group (28.42 ± 3.13%) (Figure 4d), indicating that oxygen production by the LIUS‐excited PO/D‐PGs system played an important role. In order to further verify the chemo‐sonodynamic efficacy of PO/D‐PGs system, we further studied the live/dead assay and CCK8 assay of MDA‐MB‐231 cell line under the same experimental conditions (Figure S5, Supporting Information), and the results showed a trend consistent with that of 4T1 cell experiment. More apoptotic cells could be observed in the PO/D‐PGs group, suggesting that controlled release of oxygen in the PO/D‐PGs system plays an important role in antitumor chemo‐sonodynamic therapy. Hypoxia inducible factor 1*α* (HIF‐1*α*) is a protein strongly expressed in cancer cells under hypoxia conditions, so we further examined HIF‐1*α* expression in different groups to verify that the PO/D‐PGs system improves hypoxia (Figure S6a, Supporting Information). Quantitative results showed that the LIUS stimulated PO/D‐PGs system could effectively reverse the hypoxic microenvironment and significantly reduce the expression of HIF‐1*α* (Figure S6b, Supporting Information).

**Figure 5:**
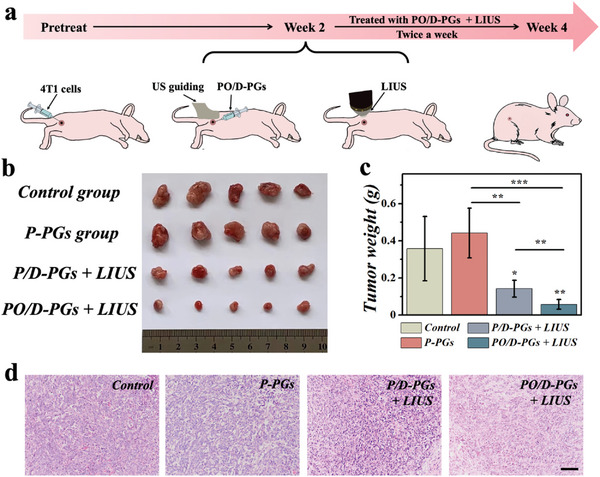
Synergistic effects of PO/D‐PGs and LIUS on murine breast cancer models. a) Schematic diagram of flow chart. b) Images captured digitally of the tumor. c) A statistical evaluation of tumor mass (*n* = 5). Data are showed as mean ± SD. *p < 0.05, **p < 0.01, ***p < 0.001, using the two‐sided student's *t* test. d) Images of various groups of tumors with H&E staining. Scale bar is 50 µm.

Encouraged by these remarkable in vitro therapeutic effects, we successively investigated the anti‐tumor efficacy of PO/D‐PGs in vivo. By implanting 4T1 cancer cells subcutaneously into the right hind leg, 4T1 tumor‐bearing mice can be obtained.^[^ ^26^ ^]^ Once tumors grew to 3–5 mm, mice were randomized into following groups: control, P‐PGs, P/D‐PGs + LIUS and PO/D‐PGs + LIUS groups. Treatments were administered twice a week (**Figure** **5**a). In the control group, tumor‐bearing mice were administered with physiological saline. After ultrasound‐guided in situ injections of pollen microcarriers for 40 min, mice in the P/D‐PGs + LIUS and PO/D‐PGs + LIUS groups, respectively received 2 min of LIUS (1.5 W cm^−2^, 1.0 MHz) (Figure S7, Supporting Information). To ensure that the microcarriers were distributed in and around the tumor, the injection was pushed slowly during needle extraction (Figure S8, Supporting Information). The body weight and tumor size of mice were recorded and updated every three days throughout the treatment. As shown in Figure 5b, in agreement with cellular experiments, P‐PGs had no antitumor effect on the breast cancer mice (0.44 ± 0.13 g). The significant antitumor effects were detected in the P/D‐PGs + LIUS group (0.14 ± 0.05 g) and PO/D‐PGs + LIUS group (0.06 ± 0.03 g) (Figure 5c). Particularly, tumors dealt with PO/D‐PGs demonstrated a significantly greater suppression effect than those treated with P/D‐PGs. The P/D‐PGs + LIUS and PO/D‐PGs + LIUS groups exhibited significant disorganization of cell structure on hematoxylin and eosin (H&E) staining (Figure 5d). Additionally, the H&E staining of the major organs of mice showed no significant changes, demonstrating the biosafety of PO/D‐PGs and LIUS (Figure S9, Supporting Information).

**Figure 6:**
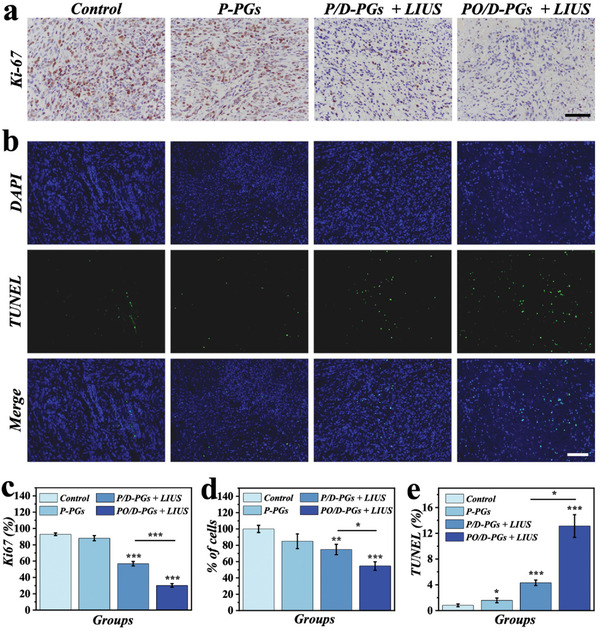
PO/D‐PGs and LIUS synergistically inhibit tumor proliferation. a) Ki‐67 immunohistochemical staining of tumor specimens. b) TUNEL staining image of tumor specimen. The statistical evaluation of c) Percentage of Ki‐67, d) Percentage of residual cancer cells, e) Percentage of apoptotic cancer cells (*n* = 3). Data in (c)–(e) are showed as mean ± SD. *p < 0.05, **p < 0.01, ***p < 0.001, using the two‐sided student's *t* test. The scale bar in (a) is 40 µm, and in (b) is 100 µm.

The multiplication and apoptosis of tumor cells was detected using Ki‐67 and Terminal Deoxynucleoitidyl Transferase‐mediated dUTP Nick‐End Labeling (TUNEL) immunohistochemical staining (**Figure** **6**a,b). Both P/D‐PGs + LIUS and PO/D‐PGs + LIUS groups showed statistically significant lower levels of Ki‐67 expression in tumor cells than the control group (Figure 6c). Moreover, Ki‐67 expression was also dramatically lower in PO/D‐PGs + LIUS group compared with P/D‐PGs + LIUS group, owing to the chemo‐sonodynamic therapy and hypoxia reversal ability of PO/D‐PGs. Immunohistochemical analysis of HIF‐1*α* expression was further conducted in different groups of tumors (Figure S10, Supporting Information). Significantly low expression of HIF‐1*α* was observed in PO/D‐PGs group. The result confirmed the antitumor efficacy of PO/D‐PGs based chemo‐sonodynamic therapy, and oxygen delivery could significantly enhance the synergistic therapeutic effect and reverse the tumor hypoxic microenvironment.

In addition, TUNEL immunohistochemical staining of tumor samples revealed that the P/D‐PGs + LIUS and PO/D‐PGs + LIUS groups contained a substantial number of apoptotic cells (Figure 6b). The proportion of cancer cells in the PO/D‐PGs + LIUS group was remarkably less than that in the P/D‐PGs + LIUS group (Figure 6d). In contrast, the quantitative TUNEL values in PO/D‐PGs + LIUS group were remarkably higher than that in P/D‐PGs + LIUS group (Figure 6e). Therefore, the good tumor suppression effect of synergistic treatment based on PO/D‐PGs + LIUS in breast cancer model indicated its promising application in the biomedical field.

## 3 Conclusion

In this study, we presented a PGs microcarrier system loaded with oxygen and chemotherapeutics in an effort to enhance the performance of chemo‐sonodynamic therapy in the treatment of TNBC. The system can be used for drug delivery and reversal of the tumor hypoxic microenvironment. Experiments using 4T1 cells and mice tumor models revealed that ultrasound‐controlled oxygen release from PO/D‐PGs can reverse the hypoxic microenvironment and produce cytotoxicity. DOX can be utilized as a chemotherapeutic drug, and also as a molecular sonosensitizer to create ROS under the influence of LIUS. In addition, the oxygen‐enriched PFC loaded in the pollen hollow cavity can release oxygen under ultrasonic irradiation, enhance the oxygen concentration and improve the ROS generation simultaneously. Consequently, the chemotherapeutics and oxygen‐carrying microcarrier system based on natural PGs provide a technique to alleviate tumor hypoxia and promote chemo‐sonodynamic therapy, which can be implemented as theoretical basis and treatment strategy for hypoxic malignancies. We believe that the cross‐state drug codelivery capacity and ultrasound‐responsiveness of the proposed nature pollen‐based delivery system shows great potential in combinational tumor treatment.

## 4 Experimental Section

### Materials

Doxorubicin (DOX) was purchased from Sangon Biotech. 1,1,1,2,3,4,4,5,5,5‐decafluoropentane was purchased from Nanjing Chemlin Chemical Industry. Sunflower pollens and oxygen were purchased from Taobao. Acetone, cyclohexane, and Sulfuric acid was purchased from SINOPHARM. Trypsin‐EDTA solution, phosphate buffer saline (PBS), Dulbecco's modified Eagle's medium (DMEM), fetal bovine serum (FBS), and penicillin/streptomycin (P/S) were purchased from Gibco. The cell Live‐Dead assay kit and CCK‐8 were purchased from KeyGen Biotech corporation. The Reactive Oxygen Species Assay Kit was provided from Beyotime Biotechnology. The Singlet Oxygen Sensor Green (SOSG) was purchased from Invitrogen. 3T3 cells and 4T1 cells were provided by the Chinese Academy of Sciences. The 7‐week female BALB/c Nude mice were bought from the Model Animal Research Center of Nanjing University.

### Processing of Pollen Grains

Pollen grains were prepared with reference to published research papers. First, sunflower pollen was mixed with water, vortexed for 5 min, and the pollen was filtered and recovered. This washing process was repeated 5 times. Then, the water‐treated pollen was resuspended in acetone solution, vortexed and shaken for 5 min, and the pollen grains were recovered by vacuum filtration twice and dried for 24 h at 60 °C. The acetone‐treated pollen was resuspended in cyclohexane, vortexed for 1 min, filtered and recovered, rinsed with acetone, and placed in a fume hood overnight. The degreasing process was repeated twice. Finally, the defatted pollen was stirred with sulfuric acid at 25 °C (300 rpm) for 1 h. The solution was allowed to cool and pollen was recovered by filtration. Pollen grains were rinsed repeatedly with deionized water until the pH became neutral. Acid‐treated hollow pollen was dried overnight at 60 °C and dried and stored for subsequent tests.

### Characterization of Pollen Grains

Analysis of field emission scanning electron microscopy for Pollen grains (UltraPlus, Zeiss, Jena, Germany). Using scanning electron microscopy (SEM), morphological alterations in pollen grains were detected following various treatments. A fluorescent microscope was used to observe pollen grain fluorescence images.

### The LIUS‐Induced Oxygen Release

By providing oxygen (99.9%) in a gas cleaning container and treating for 5 min, oxygen‐rich PFC was generated. For downstream studies, a low‐intensity Sonoplus190 (Enraf‐Nonius, Netherlands) was utilized, and the parameters were 50% duty cycle, 1.0 MHz center frequency, and 1.5 W cm^−2^ power. A dissolved oxygen monitor measured the level of dissolved oxygen in PBS (JB‐607A, Leici, China). PO‐PGs were introduced into a tube filled with 1 mL PBS to assess the oxygen carrying and releasing capacity of the PFC. The surface of PBS was treated with 1 mL of liquid paraffin to avoid gas leakage. The ultrasonic gel was utilized to separate the air between the ultrasonic probe and the side wall of the test tube. Dissolved oxygen levels were measured at regular intervals.

### DOX Release In Vitro

To investigate DOX release, the DOX‐PGS composite was added to 2 mL PBS buffer (pH = 7.4 and 5.5) and gently stirred at 37 °C. To investigate the effect on ultrasonic irradiation on the drug releasing, the composites were exposed to ultrasonic irradiation (1.0 MHz, 1.5 W cm^−2^, 50% duty cycle). Within a specific time, the original PBS was exchanged with equal amount of fresh PBS. The typical light absorbance measured DOX release at 480 nm. This experiment was repeated three times to reduce experimental errors.

### Detection of Singlet Oxygen

SOSG, a common molecular probe, was utilized to detect ^1^O_2_ generation in the system. Briefly, 5 µL of SOSG solution (5 × 10^−3^ m) was combined with 2 mL DOX‐PGs solution (DOX: 10 µg mL^−1^). At predetermined intervals, the combination was subjected to LIUS (1.0 MHz, 1.5 W cm^−2^, 50% duty cycle). Using a fluorescence spectrophotometer, the SOSG fluorescence intensity was measured. Then, the impact of oxygen on the production of singly linear oxygen was examined using the same method, such as, i.e., by replacing the DOX‐PGs solution with the PO/D‐PGs solution. In addition, a control experiment was set up under the same conditions, mixing 5 µL SOSG solution (5 × 10^−3^ m) with 2 mL pure water.

### Detection of •OH via ESR

The •OH generated by samples (with DOX concentration of 10 µg mL^−1^) under US irradiation (1 MHz, 1.5 W cm^−2^, 2 min) with different intervals was determined by an electron paramagnetic resonance spectrometer (Bruker EMXmicro‐A300). Control group included samples with pure water. For •OH detection, DMPO was added.

### In Vitro Cellular ROS Production

To validate intracellular ROS production, 4T1 cells (1 × 10^5^ cells per well) have been placed in 6‐well plates and incubated in the dark for 12 h, respectively, with control, LIUS, P‐PGs, P/D‐PGs, P/D‐PGs + LIUS and PO/D‐PGs + LIUS (DOX: 10 µg mL^−1^, 500 µL; US: 1.0 MHz, 0.5 W cm^−2^, 50% duty cycle, 2 min) were exposed for 40 min. Immediately after adding DCFH‐DA to each Petri dish, 30 min were spent in the dark environment. Finally, the production of ROS was measured by measuring fluorescence intensity in DCF cells.

### Biocompatibility Study of Pollen Grains

3T3 cells were grown in DMEM (10% fetal bovine serum and 1% P/S) in a humid atmosphere with 5% carbon dioxide at 37 °C. At the beginning of the exponential growth phase, 3T3 cells were seeded into wells of a 96 well plate at a density of 6000 cells per well. The group that received P‐PGs had 3T3 cells cultured together with P‐PG. After a 48‐h incubation, a live/dead stain was done. CCK‐8 assays were done at predetermined intervals (0, 24, 48 h).

### Cytotoxicity Assay

4T1 cells were cultured in a humid environment with 5% CO_2_ and 20% O_2_. On the outside side wall of the dish in the US treatment group, an ultrasonic transducer was placed. Using a Live and Dead Viability/Cytotoxicity Assay Kit, the 48 h viability was determined for 4T1 cells subjected to various treatments and incubations. The 4T1 cells were randomly divided into six groups, namely, control, LIUS, P‐PGs, P/D‐PGs, P/D‐PGs + LIUS and PO/D‐PGs + LIUS groups. The control group received no treatment. The LIUS group was administered LIUS for two minutes. The remaining four groups were introduced in Petri dishes with P‐PGs, P/D‐PGs, P/D‐PGs, and PO/D‐PGs. For the P/D‐PGs + LIUS and PO/D‐PGs + LIUS groups, a total of 40 min incubation was followed by 2 min LIUS. After 4T1 cells were treated for 48 h, a medium containing CalceinAM and PI was added. After 20 min in the dark, fluorescence images of these cells were obtained using a fluorescence microscope. Cell viability of treated 4T1 cells was determined using CCK‐8. 4T1 cells from each group were treated with CCK‐8 reagent (10 µL) and medium (90 µL) into each well after 48 h of culture. After two hours, they were cocultured. At 450 nm, the optical density values were measured.

### Animal Experimentation

The Nanjing University Model Animal Research Center provided twenty female Balb/c mice weighing around 20 g. All animal tests were conducted in accordance with the standards set by the Animal Ethics Committee (No.2021AE01073) of Drum Tower Hospital affiliated to the Medical School of Nanjing University.

In order to examine the antitumor effects of PO/D‐PGs in mice with mammary tumors, log phase breast cancer cells 4T1 were formed into a cell solution (1 × 10^6^ cells mL^−1^) and injected subcutaneously into the upper right hind leg of each mouse (50 µL per mouse). The tumor growth was examined by ultrasound. The mice were separated into 4 groups at random after 2 weeks when the tumor diameter was approximately 3 to 5 mm: (1) control group (100 µL saline per mouse), (2) P‐PGs group (100 µL aqueous solution of pollen grains containing PFC per mouse), (3) P/D‐PGs + LIUS group (100 µL solution of pollen grains containing PFC and DOX per mouse, 1 mg kg^−1^ of DOX), (4) PO/D‐PGs + LIUS group (100 µL oxygen‐enriched PFC and DOX solution per mouse, 1 mg kg^−1^ of DOX). 2 min‐LIUS (1.0 MHz, 1.5 W cm^−2^, 50% duty cycle) was given 40 min after injection in the PO/D‐PGs + LIUS and P/D‐PGs + LIUS groups, respectively. Relevant treatment was given twice weekly. Tumors from each group were removed and weighed after mice had been treated for 2 weeks. Mainly, organs were taken at random and treated in a 10% paraformaldehyde solution for histological investigation. In addition, TUNEL, H&E, and Ki‐67 stains were applied to sections of tumors taken from each mouse group.

### Statistical Analysis

All statistical analyses were conducted using Origin 2019b. The data are presented as mean ± standard deviation. The two‐sided Student's *t* test was used for comparative analysis. Statistical significance was determined when *p* < 0.05. And the NS signify no statistical significance, label * means *p* < 0.05, ** means *p* < 0.01, and *** means *p* < 0.001.

## Conflict of Interest

The authors declare no conflict of interest.

## Author Contributions

Y.J.Z. designed the idea and provided funding support. B.J.W. conducted the material experiments and data analysis, and wrote the manuscript. D.Q.H. supervised the animal experiments and revised the paper. C.H.S. assisted with the cell experiments. J.Y.S. checked the data.

## Supporting information

Supporting Information

## Data Availability

The data that support the findings of this study are available from the corresponding author upon reasonable request.
