# Supplementary material for: Ultrasound‐Responsive Oxygen‐Carrying Pollen for Enhancing Chemo‐Sonodynamic Therapy of Breast Cancer
Source: Adv Sci (Weinh). 2023 May 16;10(21):2300456. doi: 10.1002/advs.202300456 (PMC10375146; doi:10.1002/advs.202300456)
Supplement: Supplementary file 1 — Supporting Information [file ADVS-10-2300456-s001.pdf]

ADVANCED SCIENCE
Open Access

Supporting Information

for *Adv. Sci.*, DOI 10.1002/advs.202300456

Ultrasound-Responsive Oxygen-Carrying Pollen for Enhancing Chemo-Sonodynamic Therapy of Breast Cancer

*Baojie Wen, Danqing Huang, Chuanhui Song, Jingyang Shan and Yuanjin Zhao**

Supporting Information

Ultrasound-responsive oxygen-carrying pollen for enhancing chemo-sonodynamic therapy of breast cancer

Baojie Wen [a], Danqing Huang [a], Chuanhui Song [a], Jingyang Shan [a], Yuanjin Zhao [a,b,]*

a. Department of Ultrasound, Institute of Translational Medicine, The Affiliated Drum Tower Hospital of Nanjing University Medical School, Nanjing 210008, China

b. State Key Laboratory of Bioelectronics, School of Biological Science and Medical Engineering, Southeast University, Nanjing 210096, China

Email: yjzhao@seu.edu.cn

Supporting Figures:

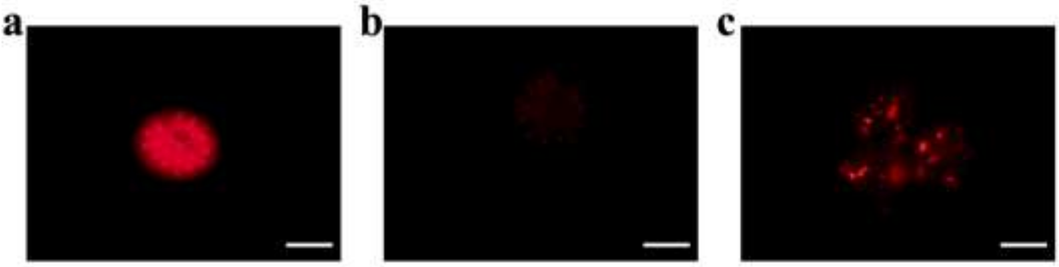

Figure S1. Fluorescence microscopy of fluorescent bleached and DOX-loaded carbonized pollen. (a) Fluorescence microscopy of carbonized pollen. (b) Fluorescence microscopy of fluorescent bleached carbonized pollen. (c) Fluorescence microscopy of DOX loaded with fluorescent bleached pollen. Scale bar is 20 μm in (a-c).

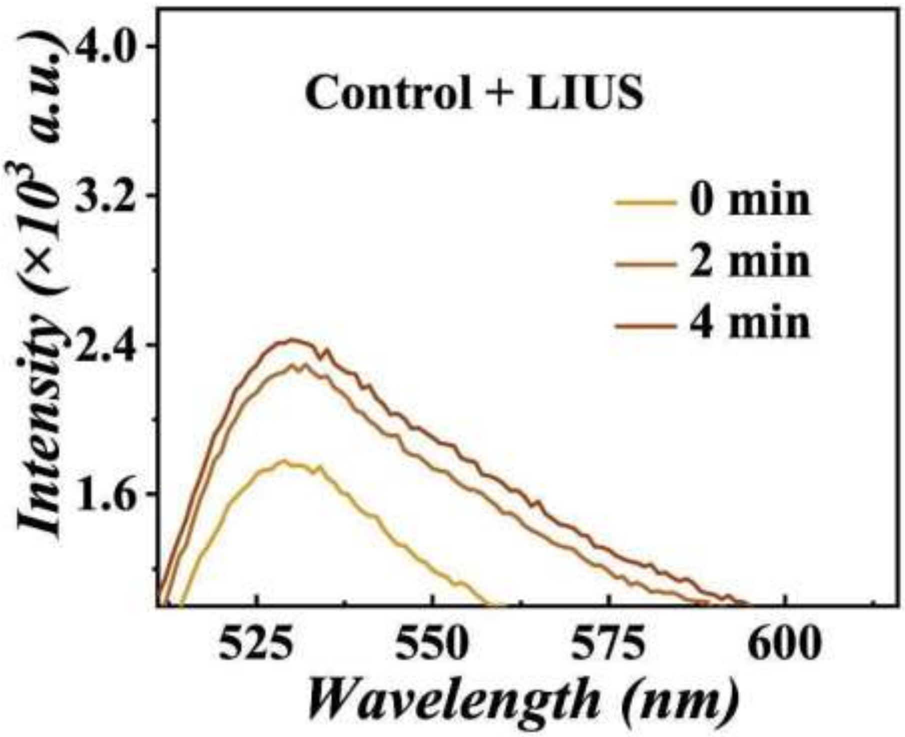

Figure S2. Fluorescence intensity curves of SOSG and pure water mixed solutions after different intervals of LIUS irradiation.

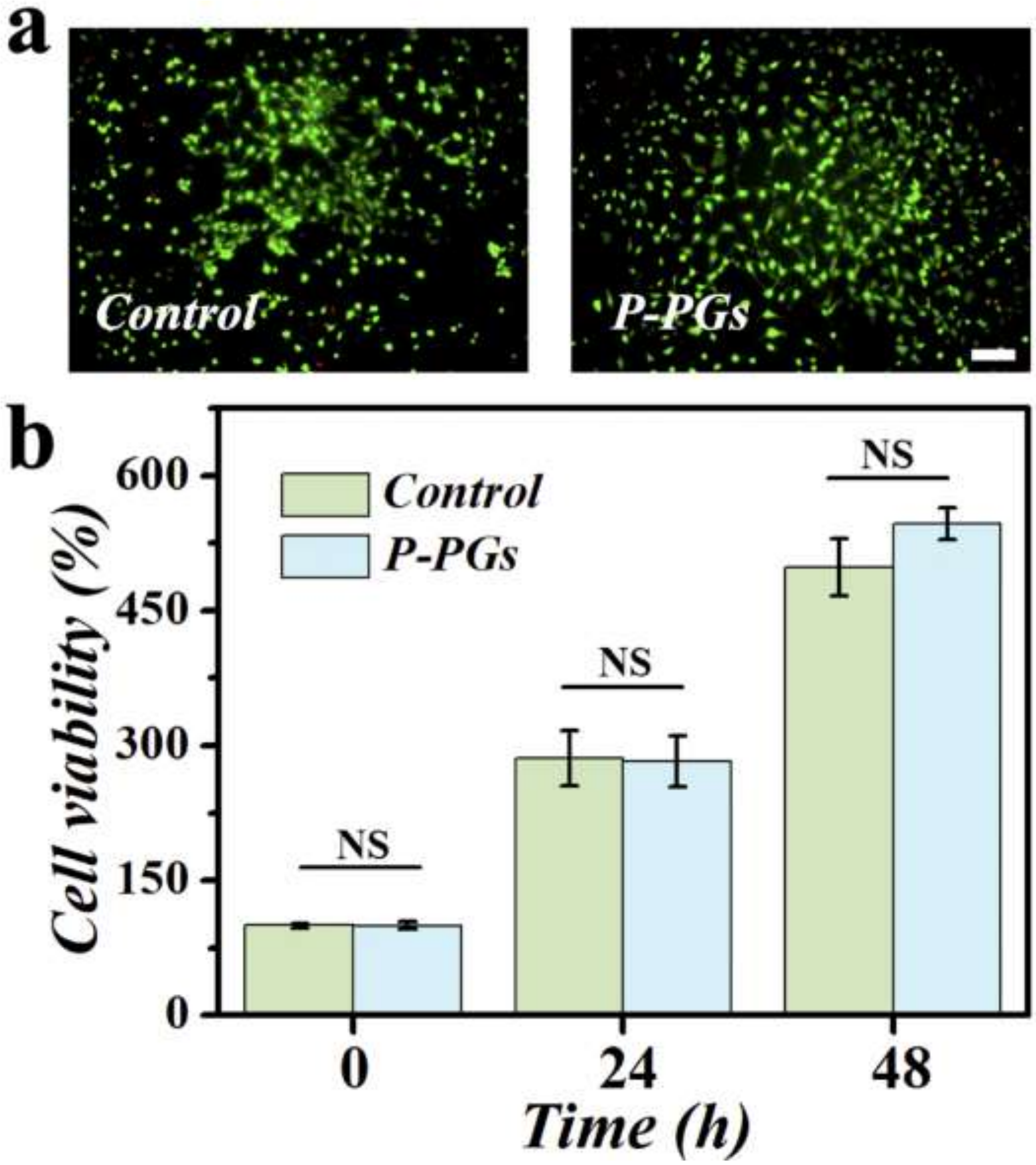

Figure S3. Biocompatibility study of P-PGs on 3T3 cells. (a) Live/dead staining images of 3T3 cells cultured in blank medium and co-cultured with P-PGs for 48h. Scale bar is 100 μm. (b) CCK-8 results of 3T3 cells at different time periods; error bars indicate standard deviation.

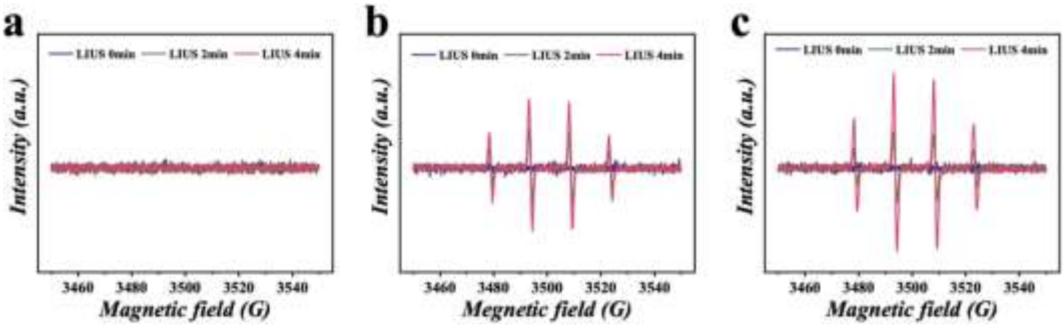

Figure S4. Detection of •OH via ESR analysis. (a) The •OH of the control group was determined after ultrasonic irradiation at different time intervals by ESR. (b) The •OH of the P/D-PGs solution was determined after ultrasonic irradiation at different time intervals by ESR. (c) The •OH of the PO/D-PGs solution was determined after ultrasonic irradiation at different time intervals by ESR.

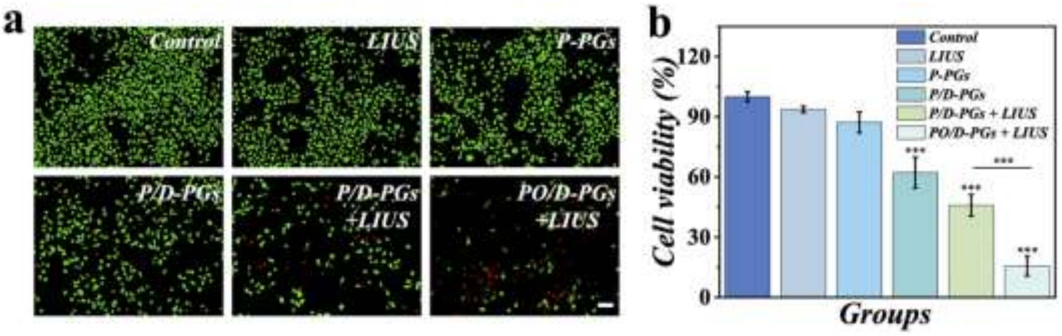

Figure S5. Cytotoxicity of MDA-MB-231 cells. (c) Live/dead staining images of MDA-MB-231 cells in different cell groups. (d) The relative cell viability of MDA-MB-231 cells in different groups (n = 6). The scale bar is 100 μm.

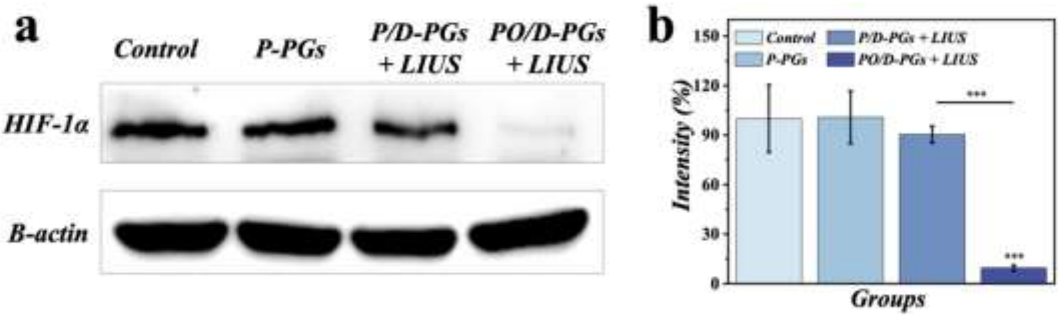

Figure S6. Western blotting assay of HIF-1α in different groups. (a) Western Blotting results of HIF-1α expression. (b) Quantification of HIF-1α expression (n = 3).

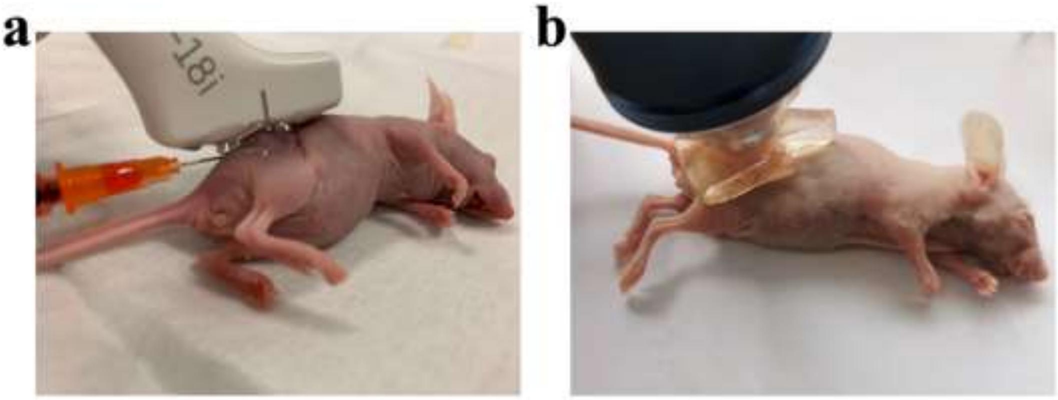

Figure S7. The recording images of the treatment. (a) US-guided intratumoral injection of pollen grains. (b) Irradiation with LIUS after injection.

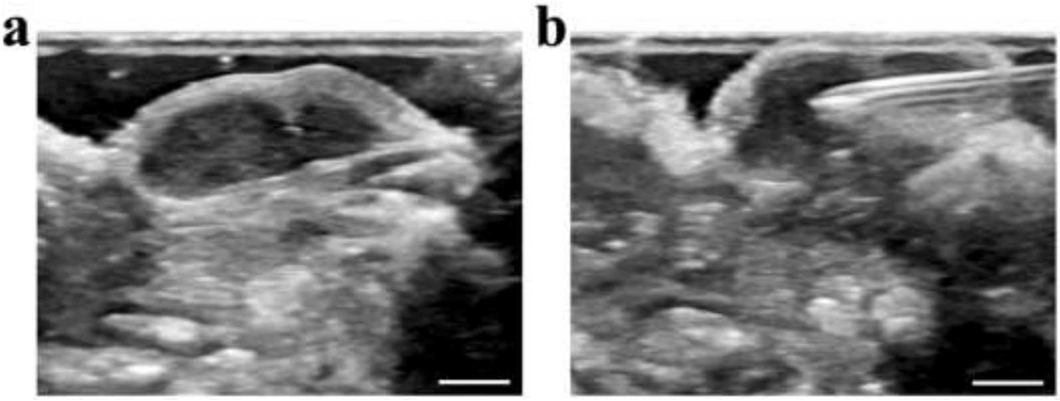

Figure S8. Ultrasound imaging of the process of injecting pollen microcarriers. (a) Grayscale ultrasound image of the tumor during ultrasound examination. (b) Ultrasound-guided needle insertion into the tumor. Scale is 2.5 mm in (a) and (b).

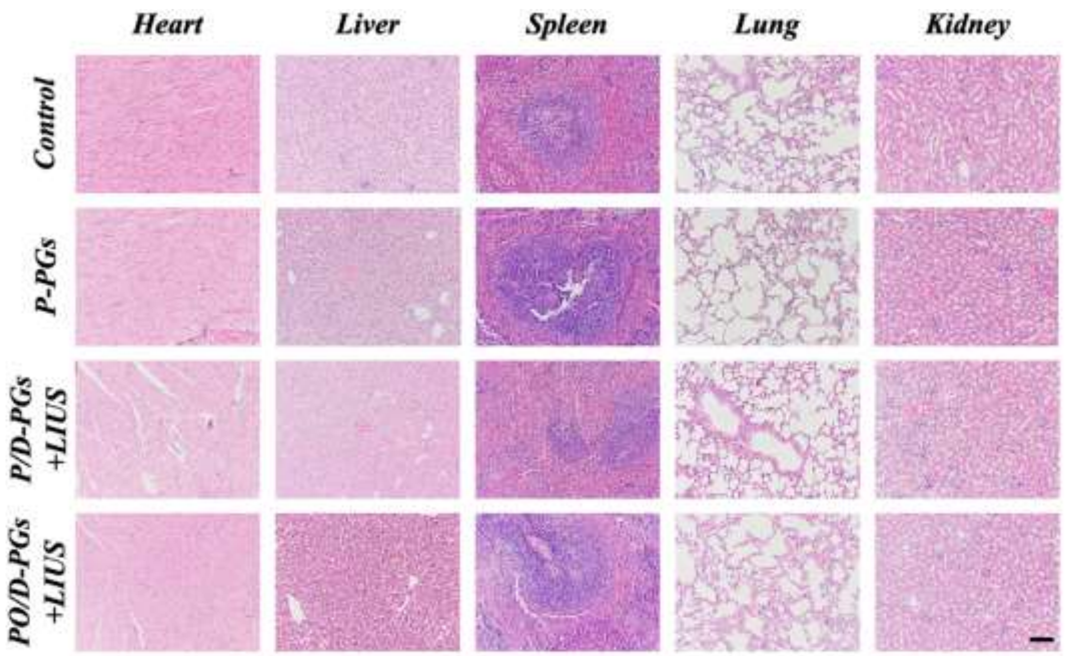

Figure S9. H&E staining of major organs of mice in each experimental group. The scale bar represents 100 μm.

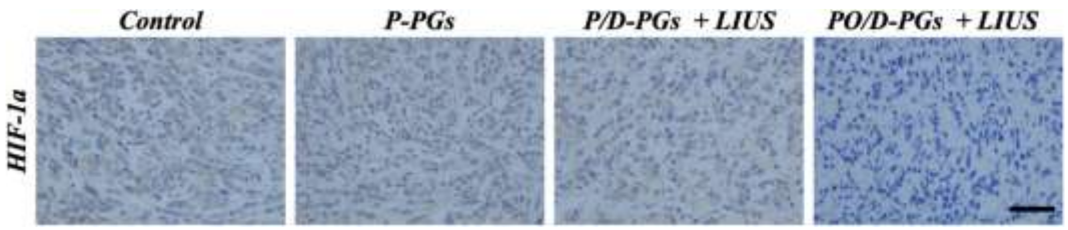

Figure S10. Immunohistochemical results of HIF-1α in different groups of breast cancer. Scale bar is 40 μm.
